# Case Report: Emergency mitral valve plasty in an unstable dog with left atrial rupture secondary to myxomatous mitral valve disease

**DOI:** 10.3389/fvets.2025.1653646

**Published:** 2026-01-12

**Authors:** Tomoki Wada, Ryosuke Takemura, Hideki Yotsuida, Misato Ohashi, Naoki Miyashita, On Uezawa, Takuya Mori

**Affiliations:** 1 Japan Animal Cardiovascular Care Team, Hishie, Higashiosaka-shi, Osaka, Japan; 2 Kinki Animal Medical Training Institute and Veterinary Clinic, Hishie, Higashiosaka-shi, Osaka, Japan; 3 National Hospital Organization Osaka National Hospital, Osaka-shi, Osaka, Japan

**Keywords:** cardiac surgery, cardiac tamponade, cardiopulmonary bypass, hemopericardium, mitral regurgitation

## Abstract

**Objective:**

To describe the medical and surgical management of recurrent left atrial rupture (LAR) in a dog with severe mitral regurgitation (MR). This report focuses on challenges encountered with pericardiocentesis, the role of hemodynamic support, and the importance of invasive blood pressure monitoring.

**Case summary:**

An 11-year-old, weighing 2.9 kg, male Chihuahua was scheduled for mitral valve plasty due to severe MR. The dog developed acute collapse and dyspnea and was diagnosed with LAR and pulmonary edema; pericardiocentesis was withheld due to relatively stable hemodynamics. Despite inotropic support and diuretics administration, a second LAR occurred the following day during transport for echocardiographic examination, with a large intrapericardial clot preventing pericardiocentesis and causing shock. Although fluid therapy and ephedrine administration improved blood pressure, invasive blood pressure monitoring and strict rest were subsequently employed to maintain a lower systolic pressure, enabling safe anesthetic induction. Under cardiopulmonary bypass, clot removal was performed. Intraoperatively, due to inadequate venous return, the heart enlarged and a third LAR occurred, necessitating repair of the rupture site with a pericardial patch under cardioplegic arrest alongside chordae tendineae reconstruction. Postoperatively, the dog developed dyspnea potentially related to lung injury, transfusion effects, or residual pulmonary edema, which resolved with medical management. Marked thrombocytopenia emerged soon after switching antibiotics but improved rapidly following corticosteroid administration and discontinuation of the suspected antibiotic. The dog was discharged in good condition and remained clinically stable 1 year later.

**Conclusion:**

This case provides a detailed account for the stepwise medical and anesthetic management of recurrent LAR secondary to severe MR. During conservative treatment, pericardiocentesis was not always effective, particularly in the presence of intrapericardial clots, which might have interfered with drainage. In the setting of cardiac tamponade, increasing pre- and after-load along with positive inotropic support appeared to contribute to temporary hemodynamic stabilization. Invasive arterial pressure monitoring allowed for stable hemodynamic control and safe anesthetic induction, underscoring its importance for preoperative management. Furthermore, the third intraoperative rupture was successfully repaired using a pericardial patch, which achieved effective hemostasis and demonstrated its potential value as a surgical option for LAR repair.

## 1 Introduction

Left atrial rupture (LAR) is a life-threatening complication secondary to severe mitral regurgitation (MR) (1–6), resulting in cardiac tamponade due to increased pericardial pressure. This can cause acute collapse, weakness, and respiratory distress (4–6). In severe cases, obstructive shock can lead to sudden death; long-term prognosis is generally poor (median survival: 26, 52, and 203 days) (2–4, 6). Pericardiocentesis can lead to rapid hemodynamic improvement in dogs with cardiac tamponade (7). However, in severe cases, large clots within the pericardium may obstruct the drainage of pericardial effusion during pericardiocentesis, preventing hemodynamic improvement (4). Since the amount of bleeding from the heart depends on the pressure gradient between the bleeding site and intrapericardial space (8), increased intrapericardial pressure can equalize these pressures, which may help control bleeding (9, 10). Therefore, pericardiocentesis for hemorrhagic cardiac tamponade may disrupt the balance between the rupture site and intrapericardial pressure, potentially promoting rebleeding (9, 11, 12). Thus, performing pericardiocentesis for cardiac tamponade caused by LAR remains controversial and depends on the specific circumstances of each case. Fluid therapy and inotropic support are potential treatment options (6.17). However, there is no established standards for when to initiate them. Broader strategies for medical management of left atrial rupture also remain unsettled.

On the other hand, Mitral valve plasty (MVP) is an established treatment of MR (13–16) and has also shown favorable outcomes in dogs with MR complicated by LAR (16). However, determining whether surgery should be performed immediately upon LAR detection is challenging. Anesthesia-induced hemodynamic collapse and bleeding are major concerns when surgically intervening immediately after rupture (16). Although the importance of preoperative medical management has been discussed, few papers provide detailed discussions about medical management of LAR.

This report presents a case of recurrent LAR in a dog. Despite medical management, the dog experienced shock due to recurrent bleeding and large clot formation. Pericardiocentesis was not possible because the clot obstructed drainage. Instead, stabilization was achieved through medical therapy, guided by invasive blood pressure monitoring and hemodynamic parameters. This allowed for safe anesthesia induction and successful surgical intervention.

## 2 Case description

An 11-year-old, weighing 2.9 kg, male Chihuahua was referred to the Japan Animal Cardiovascular Care Team for pulmonary edema (PE) caused by MR and planned MVP. Medications prescribed at the referring hospital included alacepril (1.0 mg/kg every [q]24 h), pimobendan (0.65 mg/kg q12h), amlodipine (0.11 mg/kg q12h), furosemide (1.7 mg/kg q12h), and sildenafil (1.1 mg/kg q12h). The dog had a history of syncope and dry cough with a terminal retch. Physical examination revealed normal heart rate, rhythm, femoral pulses, and mucous membranes. Thoracic auscultation revealed grade V/VI systolic murmur at the left apex. Thoracic radiography revealed severe cardiomegaly (vertebral heart score [VHS] = 13.2; reference interval [RI]: 8.7–10.7) without PE. Two-dimensional echocardiography (Vivid™ E95 Ultra Edition, GE HealthCare, Tokyo, Japan) revealed severe MR due to anterior mitral valve prolapse, with a maximum velocity of 4.45 m/s on continuous wave Doppler and evidence of a cut-off sign. Left atrial and ventricular enlargement was also observed; the left atrial-to-aortic diameter ratio was 3.32; the normalized left ventricular end-diastolic diameter was 2.23. No pericardial effusion was noted. Given concerns about increased left heart volume overload caused by sildenafil, medication was discontinued; MVP was planned.

The dog subsequently presented to an emergency hospital with acute collapse and dyspnea and was diagnosed with LAR and PE. Blood pressure averaged 101/55 (71) mmHg, based on three consecutive non-invasive measurements. Therefore, pericardiocentesis was not performed. The following morning, the dog was referred for treatment. Upon presentation, the dog was lethargic with mild dyspnea. Physical examination revealed respiratory rate, 66/min; heart rate, 130/min with weak femoral pulses; and temperature, 36.3 °C. Auscultation detected muffled heart sounds and a soft murmur. Thoracic radiography revealed mild PE and a globoid heart (VHS = 14.2; [RI]: 8.7–10.7). Two-dimensional echocardiography revealed pericardial effusion with hypo-echoic density, suggestive of a clot in the pericardial space. Based on these findings, LAR secondary to MR was diagnosed. Given hemodynamic stability, we opted for observation without pericardiocentesis. The dog was hospitalized (Day 1 of hospitalization) and treated with furosemide (2 mg/kg IV q8h), pimobendan (0.15 mg/kg IV q12h), and dobutamine (5 μg/kg/min infusion) to increase cardiac output. The dog was also managed in an oxygen chamber.

**Figure1:**
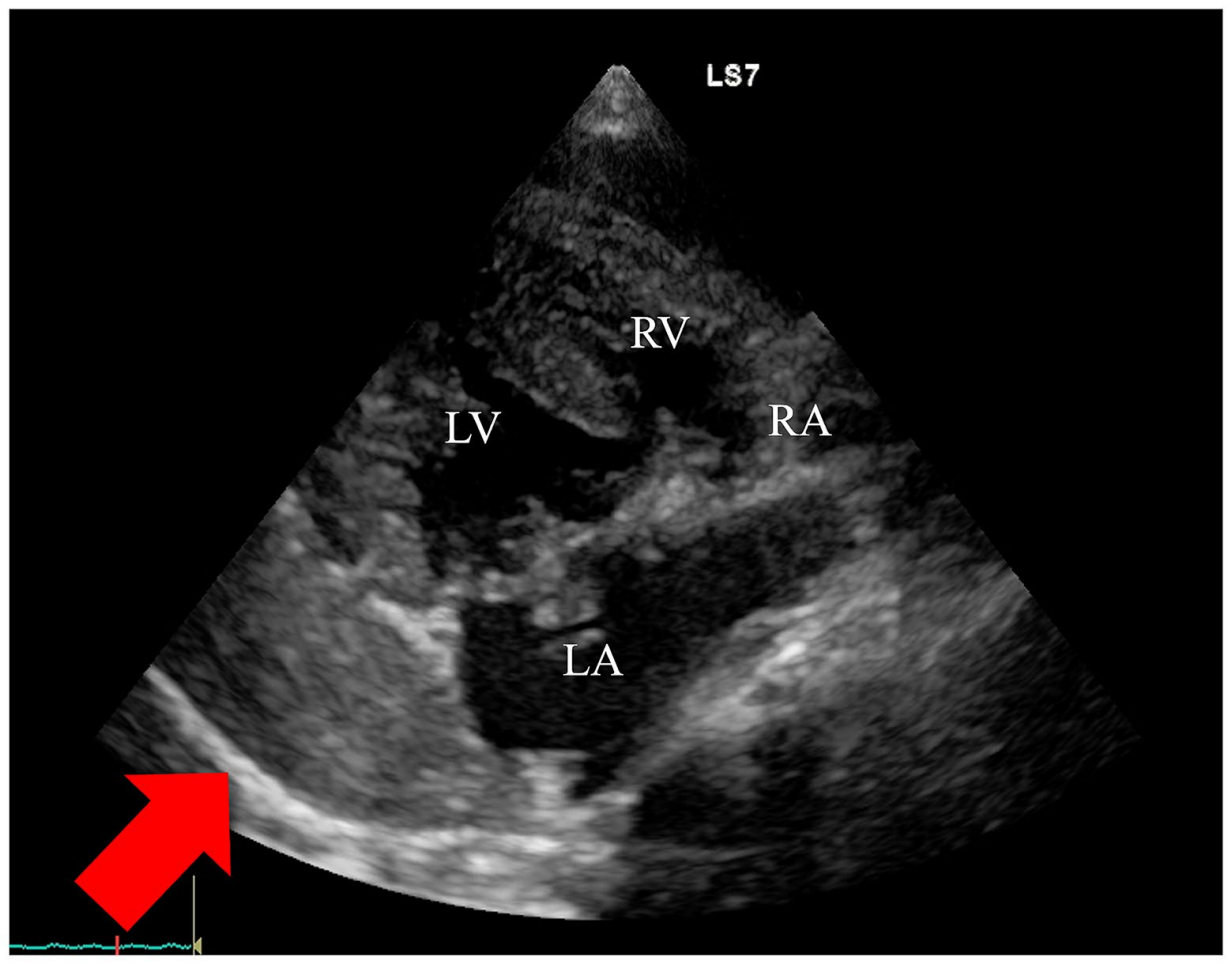
Two-dimensional echocardiography images after left atrial re-rupture on Day 2 after hospitalization, demonstrating a large clot with layered hypoechoic and hyperechoic structures, suggesting additional bleeding (arrow). LA, Left atrium; LV, Left ventricular; RA, Right atrium; RV, Right ventricular.

On Day 2 after hospitalization, the dog showed improved activity. Physical examination revealed respiratory rate, 36/min; heart rate, 150 /min with normal femoral pulses; and temperature, 38.0 °C. Although echocardiography was planned, the dog collapsed during transportation to the examination room, wherein the femoral pulses became non-palpable. The dog exhibited decreased consciousness with vertical nystagmus. Two-dimensional echocardiography revealed increased pericardial effusion and a large clot in the pericardial space (Figure 1). Pericardiocentesis was attempted from the right side at the fifth intercostal space using a catheter (22G indwelling cannula); however, the effusion was not removed due to the clot. The dog received colloid fluid (Voluven, Otsuka Pharmaceutical, Tokyo, Japan) (7 mL/kg IV) to augment preload in response to blood volume loss and ephedrine (0.05 mg/kg IV) (Nichi-Iko Pharmaceutical Co., Ltd., Toyama, Japan) to increase the blood pressure. After administration, the level of consciousness improved; however, the dog remained lethargic with a weak femoral pulse. A catheter (24G indwelling cannula) was placed in the dorsalis pedis artery to measure the arterial pressure. The recorded blood pressure (Bio-scope AM130, FUKUDA M-E KOGYO Co., LTD., Tokyo, Japan) was 79/42 (52) mmHg. Arterial blood gas (ABG) analysis (GASTAT-700, Techno Medica Co., Yokohama, Japan), while on oxygen via flow-by, revealed severe metabolic acidosis with a pH of 7.170 (RI: 7.35–7.45), decreased bicarbonate at 10.7 mmol/L (RI: 22–26 mmol/L), and a markedly negative base excess of −16.5 mmol/L (RI: −2 to +2 mmol/L), was along with hypocapnia with a partial pressure of CO_2_ of 30.5 mmHg (RI: 35–45 mmHg) and elevated partial pressure of oxygen at 236.6 mmHg (RI: 80–100 mmHg). Finally, surgical intervention, including clot removal and MVP, was then pursued. During preparing for surgery, systolic blood pressure was intentionally maintained at 80–90 mmHg to prevent left atrium re-rupture, with a target mean arterial pressure of 60 mmHg. Norepinephrine was administered at 0.1 μg/kg/min via infusion to achieve this blood pressure target; dobutamine was discontinued. Lactated Ringer’s solution was also administered at 3 mL/kg/h.

**Figure 2:**
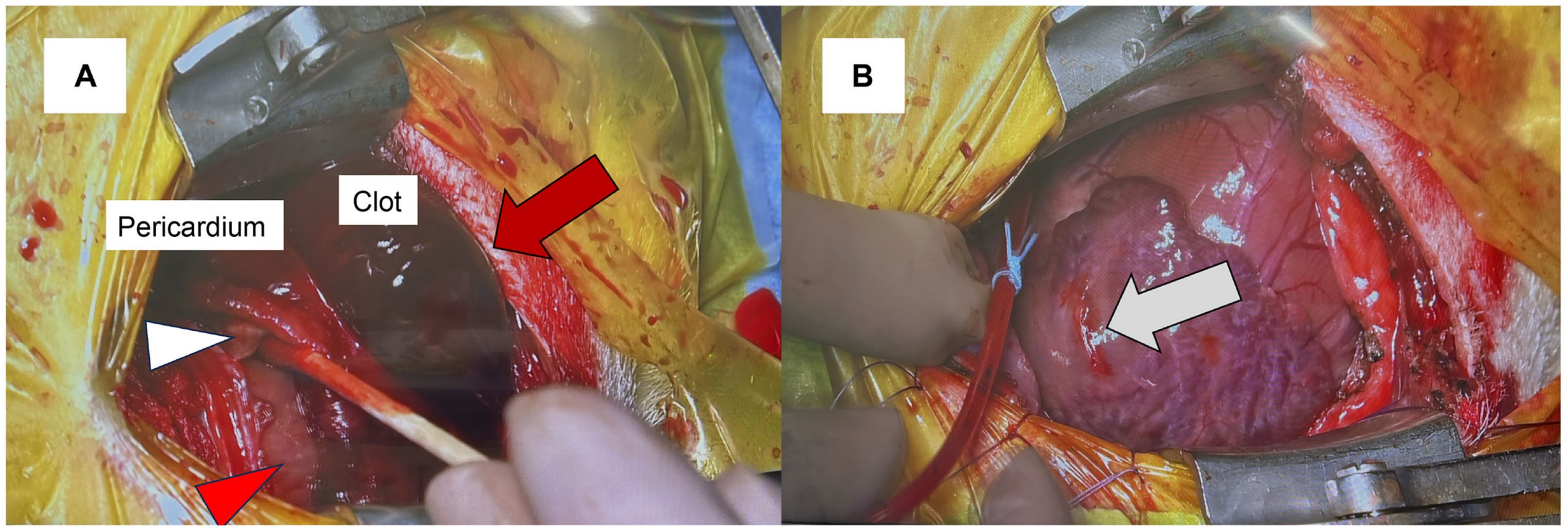
Intraoperative images showing a large clot (red arrow) resulting from left atrial rupture **(A)**. The clot was extracted from the pericardium using a swab. The white arrowhead indicates the left atrium, while the red arrowhead points to the left ventricle. A swab is inserted between the pericardium and both the left ventricle and left atrium to clear the clot. After clot removal, the site of the left atrial rupture appears as a scar (white arrow). **(B)** The clot was firmly adhered to the scar.

**Figure 3:**
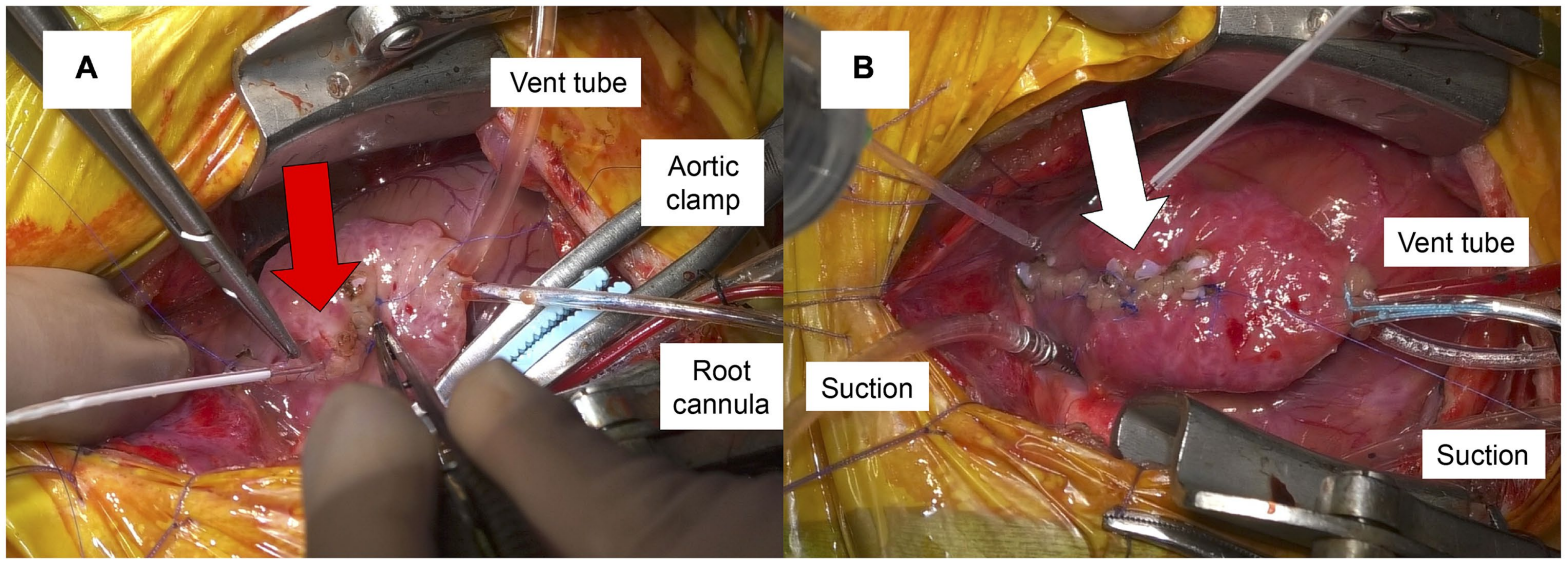
Intraoperative images of the suturing of the left atrium **(A)**. The rupture site (red arrow) was closed using pericardial pledgets and a mattress suture pattern during cardioplegic arrest. After the heart resumed beating, no bleeding was observed from the rupture site (white arrow) **(B)**.

Three hours after the second LAR, anesthesia was induced. The dog was premedicated with fentanyl (5 μg/kg IV) (Terumo Corporation, Tokyo, Japan) and cefmetazole (20 mg/kg IV) (Nichi-Iko Pharmaceutical Co., Ltd., Toyama, Japan). Propofol (Viatris, Tokyo, Japan) was administered to effect, and the dog was intubated with a cuffed endotracheal tube. No significant blood pressure fluctuations were observed during anesthesia induction. During surgery, fentanyl (2 μg/kg/h) and remifentanil (30 μg/kg/h) (Daiichi Sankyo Co., Ltd., Tokyo, Japan) were continuously infused. A left fifth intercostal thoracotomy exposed the pericardium. To prepare for potential re-rupture of the left atrium caused by pericardiotomy and clot removal, cardiopulmonary bypass (CPB) was initiated beforehand. After heparinization (400 U/kg IV) (Mochida Pharmaceutical Co., Ltd., Tokyo, Japan), a 10 French (Fr) CPB cannula (Senko Medical Instrument, Tokyo, Japan) was inserted into the left jugular vein at the level of the right atrium for the venous drainage cannula. Additionally, an 8 Fr CPB cannula (Bio-Medicus™ NextGen, Medtronic-Neurological, Minneapolis, MN, USA) was inserted into the left carotid artery for the arterial return cannula. Once CPB using a heart-lung machine (Senko Medical Instrument) was confirmed to be functional, the pericardium was incised, and the large clot was successfully removed (Figures 2A,B). Following the creation of a pericardial cradle by suspending the pericardium to improve surgical exposure, poor venous return resulted in increased blood flow back to the heart, causing sudden cardiac enlargement. Consequently, LAR occurred, leading to massive bleeding from the left atrium. The position of the venous cannula was adjusted, achieving adequate venous return, and the procedure continued with the LAR, while blood loss was suctioned throughout. An aortic root cannula (endobronchial infusion catheter, Atom Medical, Saitama, Japan) was inserted for cardioplegia administration; the heart was arrested. Chordae tendineae reconstruction and annuloplasty were performed. The left atrium was closed using 6–0 polyvinylidene fluoride (Asflex, Kono Seisakusho Co., Ltd., Chiba, Japan) with small pericardial patches created from the pericardium to reinforce the suture line and prevent tissue tearing, utilizing a continuous suture pattern. Subsequently, the LAR site was repaired using 7–0 polyvinylidene fluoride (Asflex, Kono Seisakusho Co., Ltd.) and small pericardial patches in a horizontal mattress suture pattern (Figure 3). After terminal warm reperfusion, spontaneous beating resumed. Protamine sulfate (6 mg/kg IV) (Mochida Pharmaceutical Co., Ltd., Tokyo, Japan) was administered to reverse the effects of heparinization; activated clotting time was monitored before and after reversal (pre-reversal: >400 s; post-reversal: 160 s), confirming effective neutralization. CPB was weaned after rewarming; all the cannulae and catheters were removed. A thoracic drainage tube (Atom Medical) was placed, closing the thoracotomy, while the pericardium remained open to prevent cardiac tamponade. Before anesthesia recovery, computed tomography was performed to evaluate the extent of systemic edema and check for any postoperative bleeding, revealing postoperative mild pulmonary edema. The patient was allowed to recover from anesthesia due to stable hemodynamic conditions. ABG analysis performed on room air showed a PaO_2_ of 68.8 mmHg (RI: 80–100 mmHg) immediately before transfer to the intensive care unit after extubation. The duration of cardiac arrest, CPB, and anesthesia were 100, 155, and 307 min, respectively. During surgery, a total of 50 mL of packed red blood cells and 30 mL of whole blood were transfused. After surgery, MR completely disappeared; the dog was managed in an oxygen chamber. Postoperatively, fentanyl (3 μg/kg/h) (Terumo Corporation) was administered for analgesia, carperitide (0.05 μg/kg/min) (hANP 1,000, Daiichi Sankyo Co., Ltd., Tokyo, Japan) was infused to reduce afterload and promote diuresis, and cefmetazole (20 mg/kg IV, three times daily) (Nichi-Iko Pharmaceutical Co., Ltd.) was administered as antimicrobial therapy.

On Day 3 after hospitalization (the day after surgery), the dog showed dyspnea. Due to respiratory distress, thoracic radiography was not performed at this time. ABG analysis, while in a 50% oxygen chamber, revealed a decreased partial pressure of oxygen at 43.8 mmHg (RI: 80–100 mmHg). Differential diagnoses for the dyspnea included acute lung injury induced by CPB, transfusion-related acute lung injury, and persistent preoperative PE or a combination of all of these. Following fentanyl discontinuation due to concerns about respiratory depression and the administration of prednisolone (0.5 mg/kg IV) (Riken Vet Pharma Co., Ltd., Tokyo, Japan) and furosemide (2 mg/kg IV), dyspnea improved within the day, enabling the dog to sleep and eat. ABG analysis revealed an increase in the partial pressure of oxygen to 89.1 mmHg while in a 50% oxygen chamber. Subsequently, pimobendan (0.41 mg/kg orally twice daily) (Vetmedin, Boehringer Ingelheim, Duluth, GA, USA) was administered to address postoperative myocardial dysfunction, benazepril (0.86 mg/kg orally once daily) (One Heart, MP Agro, Tokyo, Japan) to reduce afterload and provide myocardial protection, and rivaroxaban (0.35 mg/kg orally twice daily) (Xarelto, Bayer, Tokyo, Japan) to prevent thrombus formation.

**Figure 4:**
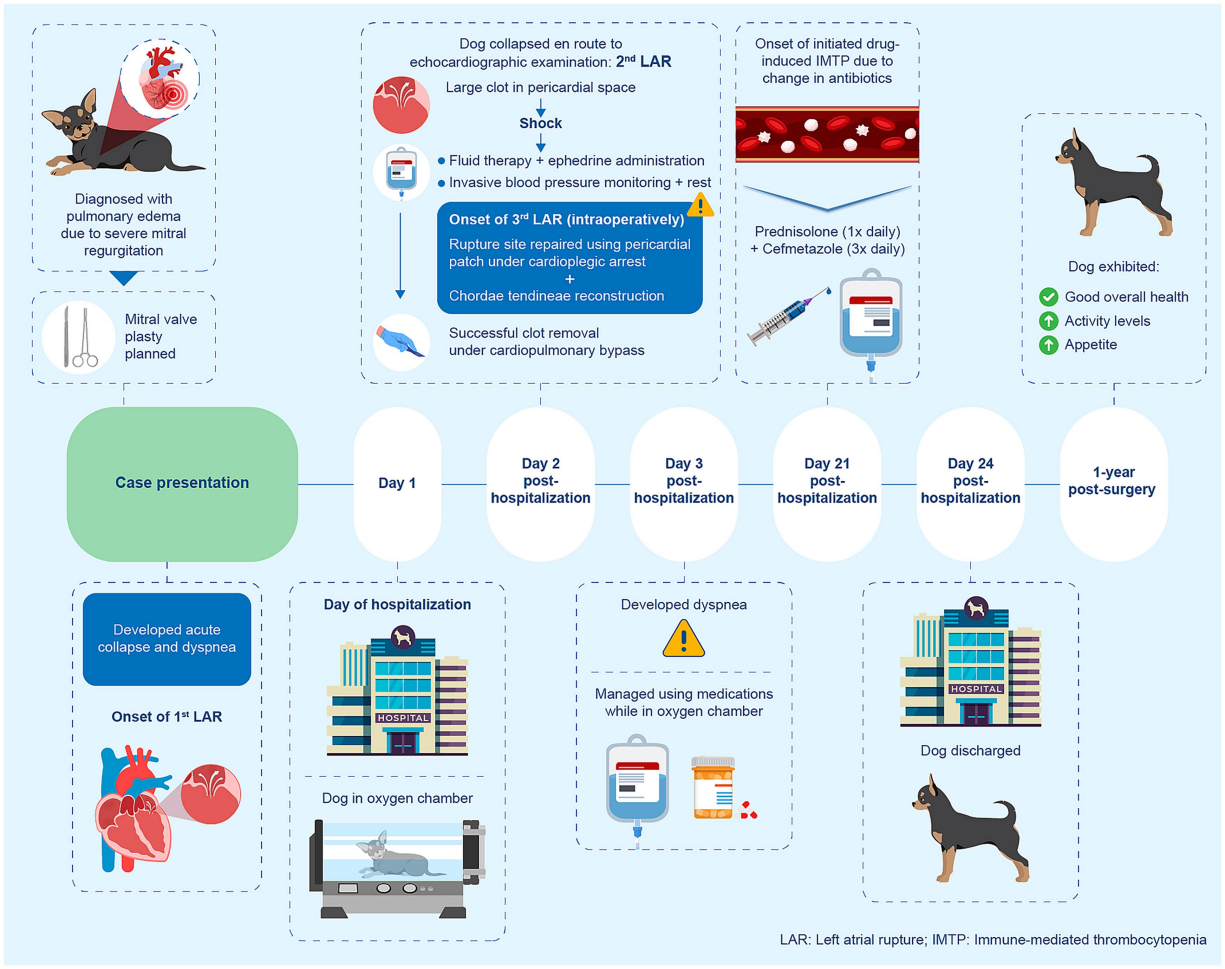
Timeline of the clinical course in a dog with severe mitral regurgitation complicated by recurrent left atrial rupture.

On Day 7 after hospitalization, significant leukocytosis was noted (46.1 × 10^9^/L; reference interval [RI]: 5.05–16.76 × 10^9^/L). Considering the possibility of a potential infection, cefmetazole was replaced with tazobactam-piperacillin (100 mg/kg IV three times daily). A blood culture was performed on the same day, revealing negative results. Since the white blood cell count gradually decreased, intravenous antibiotics were switched to oral minocycline (10 mg/kg twice daily) on Day 19 after hospitalization; discharge was planned. However, on Day 21 after hospitalization, a complete blood count (IDEXX Laboratories, Westbrook, ME, USA) revealed a marked decrease in platelets (8 × 10^3^/μL; RI: 148–484 × 10^3^/μL), with an elevated mean platelet volume of 18.6 fL (RI: 8.7–13.2 fL), compared with the previous day’s value of 234 × 10^3^/μL. White blood cell count was 13.4 × 10^9^/L (13,400/μL; RI: 5.05–16.76 × 10^9^/L [5,050–16,760/μL]). A serum biochemistry panel (IDEXX Laboratories) demonstrated an increased C-reactive protein level of 5.8 mg/dL (RI: 0–1.0 mg/dL). A second blood sample was collected, confirming a similar platelet count. No platelet clumps were observed on microscopic examination. Although no signs of bleeding (including petechiation, ecchymoses, or melena) were noted, prednisolone (2 mg/kg/day subcutaneously) was initiated considering possible drug-induced immune-mediated thrombocytopenia (IMTP). Minocycline was discontinued, and cefmetazole (20 mg/kg IV three times daily) was initiated. A rapid improvement in the platelet counts was observed shortly thereafter; prednisolone was gradually tapered and discontinued on Day 24 after hospitalization. The dog was discharged on the same day. An overview of the clinical course is illustrated in Figure 4. At 1 year postoperatively, the dog exhibited increased activity levels and appetite compared with the preoperative state and remains in good health.

## 3 Discussion

Immediate surgery is not routinely performed for LAR; conservative management is typically selected (16). However, there is no consensus on optimal medical management; few reports describe its clinical course in details (6). Potential treatments of cardiac tamponade caused by LAR include pericardiocentesis, fluid therapy, and inotropic agents such as catecholamines or pimobendan (6, 17). These options should be considered depending on patient’s condition. Although pericardiocentesis decreases intrapericardial pressure and improves the cardiac output, it can lead to rebleeding once the intrapericardial pressure is lowered and venous return increases (9, 11). Moreover, an intrapericardial clot may prevent adequate fluid drainage, rendering pericardiocentesis unsuccessful (4). Fluid therapy can enhance venous return in hypotensive patients with tamponade but must be administered cautiously because it can also promote recurrent hemorrhage (18, 19). In patients with hemorrhagic cardiac tamponade, diastolic reserve is limited, which might restrict the effectiveness of fluid therapy (20). Furthermore, hemodynamic response to fluid administration might be inconsistent due to factors such as acute or chronic nature of tamponade, baseline volume status, hemodilution effects, and the type of fluid used (e.g., crystalloids, colloids, or blood products) (20). In this case, inadequate venous drainage after pericardiotomy caused an acute increase in cardiac volume loading, which likely resulted in excessive tension on the already weakened left atrial wall and subsequently led to re-rupture. In dogs with LAR, a tear typically originates from the endocardial surface and extends into the atrial wall, producing marked structural fragility (5). Under such conditions, abrupt changes in venous return or preload can increase the transmural pressure gradient and wall stress, both of which are important triggers for subsequent rupture. Furthermore, in dogs with severe MR, an increase in preload can exacerbate regurgitant flow, causing a further rise in left atrial pressure and thereby augmenting the risk of rupture. Therefore, although pericardiocentesis or fluid therapy may transiently improve cardiac output, a sudden increase in volume loading or recurrent hemorrhage may worsen the clinical condition, necessitating careful hemodynamic management.

The role of inotropic agents in managing LAR remains unclear. Although previous experimental studies on cardiac tamponade have suggested that dobutamine and norepinephrine might be effective (20, 21), their effects in cases involving clots remain uncertain. In patients with tamponade, sympathetic tone helps maintain circulation. Therefore, cardiac output must be optimized with careful attention to several factors: preserving or increasing preload, maintaining high afterload, avoiding myocardial depression, and preventing bradycardia (22). Ephedrine, an indirect sympathomimetic, increases blood pressure by promoting peripheral vasoconstriction and raising heart rate through norepinephrine release. In this case, ephedrine was chosen for its rapid onset, and its administration resulted in an immediate rise in blood pressure. This suggests that ephedrine might be a useful option for tamponade with intrapericardial clot formation.

In this case, LAR occurred during transport for echocardiographic examination. A previous report (23) also documented LAR during echocardiography, suggesting that stress-induced increases in afterload or mechanical stress due to thoracic pressure during movement or scanning may have contributed to the condition. These additional stressors may have led to a more severe rupture than the initial episode, highlighting the importance for minimizing blood pressure elevation and ensuring strict rest. Sedation before transport may also help reduce afterload and prevent rupture in similar situations.

A previous report described surgical treatment of MR with LAR and pointed out several risks (16). The first relates to anesthetic risk. The report discontinued fentanyl preoperatively due to concerns about hypotension; careful monitoring was recommended. General anesthesia can lead to cardiovascular collapse due to reduced myocardial contractility, vasodilation, and decreased preload. Invasive arterial blood pressure monitoring is essential for managing these risks. In this case, precise blood pressure control was achieved through invasive monitoring; norepinephrine was used for supporting both afterload and contractility, which allowed for the safe administration of fentanyl. However, opioids can cause marked bradycardia and circulatory collapse, thereby having atropine prepared is advisable. Using ketamine, a drug with less cardiovascular depressant effect, might have been a preferable alternative. The second concern is rebleeding. Although early CPB initiation can stabilize circulation in patients with LAR, systemic heparinization may worsen hemorrhage. This case showed that immediate bypass is not always necessary. Invasive blood pressure monitoring confirmed stable hemodynamics, allowing proceeding with surgery using the standard MVP protocol and avoiding early heparin administration. To prepare for possible hemodynamic collapse during induction, anesthesia should be performed in the operating room with the surgeon scrubbed, the CPB team on standby; all equipment ready for immediate initiation (22). The third concern involves the left atriotomy and closure of the rupture site. In a previous report, the rupture site is included in the planned incision. However, in our case, the rupture site lay slightly away from the scheduled incision line; the tissue around the rupture was known to be friable. Therefore, we created a separate incision and closed the rupture with a pericardial patch and horizontal mattress sutures under cardioplegic arrest. Complete hemostasis was achieved, suggesting that this may be an effective approach in selected cases.

This report has several limitations. First, IMTP diagnosis was based on clinical course and response to treatment. Screening for infectious diseases and coagulation testing was not performed; platelet/megakaryocyte-associated antibody screening was not conducted due to a lack of available testing facilities. Therefore, these possibilities cannot be entirely ruled out. However, temporal association and rapid response to drug discontinuation and corticosteroids make drug-induced IMTP a plausible cause (24). Second, this is a single-case report. The effectiveness and safety of emergency surgery for LAR cannot be generalized based on one case. In this dog, postoperative complications—including respiratory distress and thrombocytopenia—were observed, making whether emergency surgery was the optimal choice unclear. Further investigation with a larger number of cases is needed to better determine appropriate timing and indications for surgical intervention.

In conclusion, we report a dog that developed shock secondary to LAR, was stabilized with medical therapy, and underwent emergency clot removal and MVP. Since few facilities can perform surgery immediately after LAR, medical stabilization plays a critical role. This case highlights a medical and anesthetic strategy centered on blood pressure control for managing LAR. From the owner’s perspective, although the sudden onset of LAR and need for emergency surgery were profoundly distressing. However, the dog’s positive response to treatment suggests that even recurrent LAR can be stabilized with appropriate care.

## Data Availability

The raw data supporting the conclusions of this article will be made available by the authors, without undue reservation.
